# Breast feeding, obesity, and asthma association: clinical and molecular views

**DOI:** 10.1186/s12948-023-00189-0

**Published:** 2023-10-03

**Authors:** Naghmeh Kian, Alireza Bagheri, Fardis Salmanpour, Afsaneh Soltani, Zahra Mohajer, Noosha Samieefar, Behzad Barekatain, Roya Kelishadi

**Affiliations:** 1 Student Research Committee, School of Medicine, Shahid Beheshti University of Medical Sciences, Tehran, Iran; 2 USERN Office, Shahid Beheshti University of Medical Sciences, Tehran, Iran; 3 Network of Interdisciplinarity in Neonates and Infants (NINI), Universal Scientific Education and Research Network (USERN), Tehran, Iran; 4 Department of Genetics, Faculty of Basic Sciences, Shahrekord University, Shahrekord, Iran; 5 Student Research Committee, School of Medicine, Ahvaz Jundishapur University of Medical Sciences, Ahvaz, Iran; 6 Division of Neonatology, Department of Pediatrics, Isfahan University of Medical Sciences, Isfahan, Iran; 7 Child Growth and Development Research Center, Research Institute for Primordial Prevention of Non-Communicable Disease, Isfahan University of Medical Sciences, Isfahan, Iran; 8 USERN Office, Research Institute for Primordial Prevention of Non-Communicable Disease, Isfahan University of Medical Sciences, Isfahan, Iran

**Keywords:** Breastfeeding, Breast milk, Asthma, Obesity, Overweight

## Abstract

Asthma is a chronic condition that affects children worldwide. Accumulating number of studies reported that the prevalence of pediatric obesity and asthma might be altered through breastfeeding. It has been proposed that Leptin, which exists in human milk, is oppositely associated with weight increase in newborns. It may also influence peripheral immune system by promoting TH1 responses and suppressing TH2 cytokines. Leptin influences body weight and immune responses through complex signaling pathways at molecular level. Although previous studies provide explanations for the protective role of breastfeeding against both obesity and asthma, other factors such as duration of breastfeeding, parental, and prenatal factors may confound this relationship which requires further research.

## Background

The role of breastfeeding in changing the prevalence of diseases has been under investigation in recent years. Breast milk includes hormones, antibodies, anti-inflammatory cytokines, and probiotic bacteria, making it the finest nourishment for infants. Breastfeeding has been demonstrated in various studies to have numerous merits, the most prominent of which are the reduction of infection and also the risk of asthma and obesity [1, 2]. Asthma, a chronic airway obstruction, is the most frequent chronic condition in children and the leading cause of pediatric hospitalization [3, 4].

Breastfeeding protects against childhood obesity. According to recent studies, breastfed infants have been more overweight than non-breastfed infants, and breastfeeding was shown to reduce newborns’ weight by 4% after a month [5–7].

Exclusive and long-term breastfeeding has also a protective role against asthma [8]. There has been a considerable reduction in wheezing and viral respiratory infections, which can contribute to asthma, especially in the early years of life. Children who have been breastfed for more than 12 weeks had a lower incidence of asthma [9, 10].

Obesity is one of the several variables that contribute to asthma. Overweightness and obesity, which have become important public health concerns in recent decades, have been associated with asthma, especially in the early years of life. Obesity-related asthma is more common in obese children who have increased amounts of inflammatory factors and reduced capacity of lungs. In children with corticosteroid-related or exercise-induced asthma, higher rates of obesity is also associated with inflammatory factors overproduction [11, 12].

There are various studies on the effect of breastfeeding on obesity and asthma, with mainly positive and acceptable results. However, few researches on the effect of breastfeeding on obesity-related asthma have been conducted. The aim of this review study was to investigate the connection between these three elements—breastfeeding, obesity and asthma- of pediatric patients. Furthermore, a precise overview of leptin signaling pathway linked to this association has been provided.

### Breastfeeding and childhood obesity association

A number of studies have suggested breastfeeding as a protective factor against childhood obesity. The risk of obesity in breastfed children has reported to be 22–24% lower than in children who were never breastfed. The effect is stronger among boys than girls [13–21]. Another study reported that the prevalence of obesity in breastfed children was 2.8%, comparing with 4.5% in those who had never been [22]. Having said that, results from several studies found no association between breastfeeding and childhood obesity [23–25].

There are inconsistent results about the associations between duration of breastfeeding and childhood obesity. Some studies demonstrated that duration of breastfeeding and childhood obesity follows a marked dose—response relationship; in other words, lowest likelihood of overweightness has been found in children with the longest duration of exclusive breastfeeding, given that the child should have been breastfed for at least 6 months. Some other studies, however, concluded that the relationship between breastfeeding duration and obesity in children is not always dose–response related [14, 15, 26–28]. In fact, one study showed that breastfeeding follows a dose–response pattern if children were fed by their mother’s milk for at least 4 weeks [29]. A cross sectional study showed that the prevalence of childhood obesity in children who were fed exclusively with breast milk for 2 months was 3.8%; the prevalence was declined to 2.3%, 1.7%, and 0.8% for children who were breastfed for 3–5, 6–12, and more than 12 months, respectively. Based on this study the dose–response pattern was present in the link between breastfeeding duration and the obesity prevalence [22].

The literature provides some explanations for the relationship between breastfeeding and childhood obesity, including confounding and physiologic factors. Confounding factors such as maternal weight age and also educational and socioeconomic statuscould explain the association between breastfeeding and childhood obesity. For example, in industrialized countries, mothers who have breastfed their children are in most cases more educated, wealthier, and older and, they have more social support for breastfeeding. Therefore, they would have a healthier lifestyle which includes regular physical activity and healthier diets, both of which leading to reduction of obesity in children [30–32]. Breast milk has a moderate amount of calories and nutrients for infant, while formulated meals has higher level of fat and protein [33, 34]. Breast milk could also affect proliferation and differentiation of the infant’s adipocytes by bioactive substances like leptin and ghrelin. Leptin is a hormone in breast milk and is generated by mammary epithelial cells [35–37].

A prospective cohort study which involved 4325 singletons between 9 and 10 years of age reported that breastfeeding for more than 6 months may reduce the risk of overweightness in children. In addition, breastfeeding was linked with lower risk of having a fat mass -measured by X-ray devices- but there was no evidence for the association between breastfeeding and obesity based on BMI thresholds [38]. Accordingly, breastfeeding for < 3 months has low protection for childhood obesity, and breast-feeding for ≤ 7 months has a significantly high protecting effect [21]. In contrast, the results of another study failed to show a certain threshold for this protective effect in which the risk of obesity was reported to be reduced by full breastfeeding for 3 months or even 6 months or more [39]. Similarly, another study reported that breastfeeding for less than 4 months has no effect on childhood obesity [40]. Diverse study populations (with different genetic and environmental backgrounds) and different sample sizes may explain the inconsistency among studies [21].

Recent studies indicated that cofactors like smoking during pregnancy may affect the association between breastfeeding duration and childhood obesity. After adjusting confounders and other known obesity risk factors, smoking during pregnancy and childhood obesity were shown to be linked, hence making smoking during pregnancy an independent risk factor for obesity in children. Smoking has been associated with a poor diet (consuming less fresh foods and more fatty foods) and lower levels of exercise. Noteworthy to say, maternal dietary factors have significant effect on obesity in children [38, 41–44]. Additionally, result of the study conducted on rats showed that exposing rats to tobacco and nicotine caused appetitive learning and attentional deficits; these behaviors are under the control of cholinergic and catecholaminergic neurotransmitter systems of the brain, so in children who have been exposed to nicotine in utero, these behaviors are being less controlled by neurotransmitters than other children [45–47].

Authors declared that early feeding could not be an independent predictor of childhood obesity and that parental and prenatal factors have significant effects, and should be considered in researches [48]. According to a prospective, observational study by Jennifer L Baker, there is a link between maternal pre-pregnancy BMI and weight gain in infancy of their children. Therefore, future investigations which assess the effects of breastfeeding on infants’ weight gain should consider this factor [49].

However, studies have shown that moderating familial factors (such as dietary habits and physical activity), screening genetic and environmental factors are more effective than breastfeeding on childhood obesity [39, 50].

### Obesity and asthma association

Obesity and asthma are both chronic inflammatory diseases that impair one’s quality of life. Asthma and obesity are caused by genetic and environmental variables such as nutrition, physical activity, and allergen exposure [51, 52]. In industrialized and developed countries, in particular, obesity and asthma are considered as important pediatric health conditions. Obesity has been more prevalent in children in recent decades. The prevalence of asthma is 20% greater in obese and overweight children [51].

Obesity was identified as a risk factor for childhood asthma by the Centers for Disease Control and Prevention (CDC) in 2015 [51]. The “obese asthma” phenotype is the term given to this condition. There are two forms of this phenotype: late-onset and early-onset [53, 54]. The early-onset type is more common, it causes obesity, and it is strongly associated with atopy which is a genetic tendency to produce high levels of IgE, and to develop allergic conditions. Obesity causes the late-onset phenotype in children above the age of 12, and it is not connected with atopy. According to a study by Attanasi [55], the late-onset phenotype accompanies dyslipidemia and insulin resistance.

According to a study by Khalid [53], asthma symptoms worsen at puberty in overweight or obese girls. Obesity is one of the factors contributing to the rise in sex hormone levels in blood. The earlier puberty begins, the more signs and symptoms we observe. Children who are overweight or obese have a 50% higher incidence of asthma. This is especially true for asthmatic individuals on medication [56, 57].

Overweight children with asthma have more clinical symptoms and attacks in a year and have a higher rate of hospital emergency visits; they require more mechanical ventilation, stay in hospital for longer time, respond less to inhaled corticosteroids, show more resistance to oral corticosteroids, and have a lower overall quality of life [58–65].

On the other hand, overweight or obese girls have a higher chance of acquiring asthma than boys, according to Deng X study [66], which is a meta-analysis of 18 studies investigating obesity and asthma in children. This study demonstrated that overweight children have a 20 percent increased risk of asthma, whereas obese children have a 49 percent increased risk. Body Mass Index (BMI = kg/m^2^) is the most accurate measure to determine whether someone is overweight or not. A BMI of more than the 85th percentile in children is considered overweight, while a BMI of more than the 95th percentile is termed obese [67, 68]. As asthmatic children are less physically active and take variable amounts of corticosteroids comparing to the children of their age, many of them are at risk for weight gain and obesity [69, 70].

Land J’s study [71] revealed that 25% of children with asthma are presently obese or overweight, and overweightness and obesity are still one of the major causes of asthma following definitive confirmation by spirometry.

Gastroesophageal Reflux Disease (GERD) and Obstructive Sleep Apnea (OSA) are the two major diseases associated with asthma and obesity. One of the causes that aggravates asthma symptoms is GERD. Obese children are more likely to develop GERD [72]. Obesity also increases the risk of OSA, which is a risk factor for asthma [73].

Truncal obesity puts pressure on the diaphragm and reduces the lung capacity [74, 75]. Following the deposition of adipose tissue in the abdomen and the increase in intra-abdominal pressure and subsequently on the diaphragm in obese children, lung volume and tidal volume are decreased. Strunk R.C [76] estimated that a rise in BMI per unit in asthmatic children lowers the FEV1/FVC (a ratio of forced expiratory volume in the first second to the forced vital capacity) and FEV1 ratios, resulting in increased airway obstruction. Obesity causes greater wheezing and orthopnea, as well as lower ERV and residual functional capacity (FRC) [77].

The body’s adipose tissue functions as an endocrine gland, and the more adipose tissue there is, the more inflammatory the body becomes [73, 78–80]. Obesity increases inflammatory adipokines such as leptin while decreases anti-inflammatory adipokines such as adiponectin [74, 75]. According to several studies, the higher the leptin, the lower the FEV1 / FVC and allergic inflammation, and vice versa [81–83]. Various studies have shown that increasing the concentration of adiponectin reduces hyper-reactive airway and reduces the severity and duration of asthma attacks in patients with asthma [84–86].

A study by Cholakovska [87] examined 72 children aged 7–15 years in three groups in terms of obesity and asthma. In overweight or obese children with asthma, eosinophilia, increased CRP and fibrinogen, and a positive prick test were reported.

A hyper-reactive airway is caused by a decrease in the suppleness of respiratory smooth muscle cells, as well as an increase in cell resistance and bronchial regeneration. Insulin resistance has been proven in several trials to increase lung function and airway hyper-reactivity [82, 88, 89].

Increased mobility, a regular exercise regimen, and a suitable diet are the best treatments for obesity in children with asthma. Anti-obesity medications like orlistat and metformin are not indicated for the treatment of obesity in children [90]. It is recommended that overweight or obese children with asthma increase their physical activity, follow good dietary guidelines, and take vitamin D supplements. Some asthmatic children who are overweight or obese do not respond well to bronchodilators. It is best to consider a leukotriene receptor blocker such as monteleukast in this group of patients [63].

In Willeboordse M’s study [91], 87 overweight or obese asthmatic children aged 6 to 16 years were placed into two groups. One group followed a diet and exercise program for 18 months. In the intervention group, improvements in lung vital capacity (FVC), quality of life, and asthma control were observed. According to another research [92], a 10-week weight-loss program reduced asthma symptoms and lung function in children aged 8 to 17. According to A study by Lucas J [93], 12 weeks of moderate intensity exercise, each session lasting 40 min, resulted in a lower BMI, increased VO2max, and improved cardiopulmonary function.

In obese children with asthma, omega-3 fatty acids are involved in improving asthma symptoms. Also, if omega-3 treatment is started before exercise, it will prevent some exercise-induced asthma [94, 95].

Several studies on the effects of vitamin D on obese children with asthma have shown that an average of 82.5% of obese or overweight children suffer from vitamin D deficiency. One of the hypotheses in this regard is that in overweight people, due to the presence of more adipose tissue, more vitamin D is deposited and its absorption is impaired [96–98]. Several studies have shown that low-dose, short-term treatment with vitamin D can reduce the severity and duration of asthma attacks and increase the response to inhaled steroids; However, there is insufficient evidence for the effectiveness of vitamin D in the management and treatment of asthma [82, 88, 89].

Increased mobility and weight loss in people with obesity and asthma may reduce the number of asthma attacks and symptoms [99]. Some studies do not fully support this hypothesis. Based on a systematic review study by Okoniewski [100], that examined a number of RCTs on the effect of weight loss in children and adults with asthma, no significant improvement in forced expiratory volume in one second (FVC or FEV1 / forced vital capacity (FVC) or expiratory reserve volume (ERV) was reported in the intervention groups compared to controls [92, 93]. The intervention groups had lower levels of leptins, IL6, IL8, TNF -α, and CRP. All therapies resulted in significant improvements in asthma control [100].

There is a continuum of endotypes for asthma, ranging from a Th2-driven (type 2) endotype to a non-Th2-driven endotype [101]. Th2-driven asthma is defined by traditional type-2 related variables, such as Th2 cells, eosinophils, and IgE. It includes early-onset allergic asthma, late-onset eosinophilic asthma, and exercise-induced asthma [102]. Non-Th2 asthma, which is associated with severe asthma and is characterized by Th1, Th17 cells, and neutrophils, comprises neutrophilic asthma and obesity-related asthma [90, 103]. Asthma-obesity endotypes may be related to age of onset [104]. Non-T2 endotypes with mixed Th17/ILC3 (type 3 immune response) and Th1/ILC1 (type 1 immune response) profiles, both of which are generated by leptin, are the principal drivers of obesity-linked asthma in adulthood [105]. A summary is provided in Table 1.

**Table 1:** The controversial association between breastfeeding and childhood obesity

Study	Country	Years	Type of study	Number of participant	Supporting or declining association
Verstraete et al. [13]	United States	2014	Cohort	196	Breastfeeding for more than 1 year has a protective effect on the development of obesity in early childhood
Li et al. [199]	United Kingdom	2003	Cross sectional	2584	No evidence was found to show that breast feeding influenced BMI or obesity
Anderson et al. [14]	United States	2013		15,141	Shorter durations of breastfeeding is correlated with the prevalence of early childhood overweight and obesity
Jwa et al. [16]	Japan	2014	Cohort	Boys = 21,425 girls = 20,147	Breastfeeding, even for short duration, provides a protective effect against obesity in late childhood, especially for boys
Von Kries et al. [200]	Germany	1999	Cross sectional	9357	Breastfeeding might reduce obesity in childhood and consequently adulthood
Burdette et al. [201]	United States	2006		313	Breastfeeding and the timing of the introduction of complementary foods were not associated with adiposity at age 5 years
Gillman et al. [202]	United States	2008	Cohort	1,110 mother–child pairs	The lower risk of overweight at age 3 years is correlated with healthful levels of maternal smoking during pregnancy, gestational weight gain, breastfeeding duration, and infant sleep duration
Kwok et al. [25]	Hong Kong	2010	Cohort	7026	Breastfeeding was not shown to be associated with child adiposity
Kramer et al. [27]	Canada	2007	Randomized Controlled Trial	17,046	Breastfeeding did not result in reducing the measures of adiposity in healthy breastfed infants

### The impact of breastfeeding on asthma

Breastfeeding has a protective effect against immunological disorders, although the mechanism by which it does so is still unknown. Certain factors of breast milk, such as immunoglobulins, growth factors, and vitamins, help the body preserve the stability of the intestinal mucus barrier, thus making it important for the development of a tolerogenic immune response in children [106, 107]. The most common immunoglobulins in human breast milk are IgA antibodies, which protect the newborn from infections and atopic disorders [108]. In addition to the so-called factors, there are plenty of anti-inflammatory cytokines in breast milk, including gamma interferon (IFN-γ), transforming growth factor (TGF-β), and interleukins such as IL-6 and IL-10. These components have been demonstrated to mitigate the negative effects of toxic stress [109].

A hypothesis suggests that early respiratory infections play a role in the development of later asthma, thus addressing the question as to why the protection offered by breastfeeding continues to stand out in adulthood [2, 110, 111]. According to studies, breastfeeding for extended lengths of time, makes children less prone to developing asthma. The reduction in the risk of acquiring asthma is especially noticeable in children under the age of two. The significance of the effect weakens as children grow older. This is also in accordance with the findings that suggest during the first 6 months of life, breastfeeding is linked to a reduced risk of hospitalization due to infectious diseases such as gastrointestinal or respiratory disorders, which cause wheezing conditions. There has been a dose-dependent pattern to this relationship [112–114]. In 2008, an investigation by the American Academy of Pediatrics (AAP) determined that exclusive breastfeeding for at least 12 weeks protects children from wheezing at an early age. Also, in the first 2 years of life, continuous breastfeeding, but not exclusive, is protective against wheezing conditions [115]. Additionally, exclusive breastfeeding for less than 12 weeks is strongly linked to overweightness [10, 116]. Because several factors, including gender, parental asthma, passive smoking at birth, and residence location might affect respiratory disorders, it becomes more difficult to identify the precise effect of breastfeeding on this subject as individuals age. Although some subsequent research has yielded inconsistent findings, because of cultural differences and varying levels of economic development, breastfeeding culture and its impact on respiratory diseases, as well as prevalence and causes of asthma, could also differ from country to country [117]. As a result, the International Study of Asthma and Allergies in Childhood (ISAAC) divided its findings into Western and non-Western countries. According to one ISAAC study, breastfeeding has been linked to a lower risk of wheezing in both wealthy and impoverished nations, but only for non-atopic wheezing in impoverished countries [118]. Overall, children who were exclusively breastfed for less than 12 weeks were more likely to be diagnosed with asthma. Furthermore, exclusive breastfeeding for less than 12 weeks was strongly related to overweightness in children aged 8 to 10. As shown by research, children who were exclusively breastfed and overweight at the time of the study, were more prone to developing asthma, compared to children who were exclusively breastfed for a longer period and were not overweight. In addition, children who were either exclusively breastfed for less than 12 weeks or who were overweight had a moderate recurrence of asthma. However, the chances were not considerably higher than the children who did not have any of these statuses, which indicates that being either overweight or breastfed for less than 12 weeks alone is not substantially linked with asthma. Studies have shown that the relation between overweightness and breastfeeding condition and developing asthma is substantial in patients whose mother has been diagnosed with asthma. Also, compared to girls, the relation is stronger in boys [119]. Despite the fact that the pathophysiology is yet unknown, the WHO and the AAP promote breastfeeding as the first choice of nourishment for newborns [120, 121].

*The impact of breastfeeding on obesity-related asthma* The link between obesity, asthma, and a short period of exclusive breastfeeding indicates a shared mechanism, which could be mediated by leptin [122]. The leptin level is higher in children who were breastfed. Leptin inhibits food intake and so controls body weight via affecting the hypothalamus [123]. Leptin levels in serum are oppositely associated with weight gain in newborns [124]. Furthermore, research suggests that leptin influences the peripheral immune system by promoting TH1 responses and suppressing TH2 cytokines. Boys have lower levels of leptin in umbilical cord blood and during the first years of life than girls, which could contribute to delayed TH1 immunological responses in boys, increasing their risk of being overweight [125].

It is noteworthy that breast milk and maternal nutritional diet can alter the infant’s intestinal microbiota. Recent studies have found that obesity-related maternal intestinal microbiota can cause changes to the infant intestinal microbiota towards dysbiosis, which might ultimately result in obesity [126–129]. Additionally, evidence indicating obese breastfeeding mothers had milk with a considerably less diverse microbiome supports the idea that obesity affects the microbiota [130–133]. In the milk produced by obese mothers who maintained high-fat diets throughout pregnancy and lactation, the quantity of the Bacteroides genera was drastically reduced [131, 134]. Obesity changes the balance of the mother's microbiome during pregnancy and lactation, which could significantly affect a child's chance of developing asthma in the early years of life [126]. Alterations in the microbiota balance cause reactions from the immune system. When the intestinal microbiota of infants of obese mothers is deficient in Proteobacteria spp., the immunological profiles may change, increasing the likelihood of acquiring inflammatory conditions [135]. Mothers who were obese or maintained a high-fat diet had infants with an imbalanced intestinal microbiota. This imbalance in the microbiota could not be completely amended after receiving a specialized diet in the form of complementary feeding. This shows that altering a mother's diet can alter her intestinal microbiota, which can eventually lead to a long-term imbalance in the infant's intestinal microbiota. These modifications may put the child at a high risk of inflammatory diseases such as asthma [129]. Furthermore, compared to breastfeeding, formula feeding carries an increased risk of obesity and asthma in a child [136]. Infants who are formula fed are provided with a wider range of nutrient content and more complex types of carbohydrates than those who are breastfed. This encourages the colonization of a variety of intestinal microorganisms [137]. Consequently, knowing how the milk microbiome affects obesity-related asthma may help researchers find innovative ways to modify diets both before and after child delivery to lower the chance of developing asthma.

## A comprehensive review of leptin signaling pathways

### Leptin signaling pathway in the hypothalamus

The central nervous system (CNS), which controls both food intake and energy expenditure, is essential for preserving the homeostasis of the body's total energy. The hypothalamus, one of the brain's regions, is crucial in the regulation of energy balance [138]. The most important hormone in the homeostatic control of energy balance is Leptin, a 16-kDa polypeptide that is encoded by the *ob* gene. It largely controls blood glucose levels, thermogenesis, and hypothalamic neurons to control food intake [139–141]. Obesity is caused by a leptin deficit or genetic flaws in the leptin signaling pathway's constituent parts. Leptin receptor b (LEPRb)-expressing neurons in the brain, notably in the hypothalamus, are primarily responsible for leptin's regulation over energy balance and body weight [142].

### Leptin receptor in the hypothalamus

The hypothalamus in particular is thought to be the primary leptin target that causes leptin's anti-obesity activity in the CNS [143, 144]. The long form of leptin receptors (LEPRb), which is present in various parts of the brain, is where leptin performs its biological activity by attaching to and activating it [145, 146]. A member of the family of cytokine receptors of the interleukin 6 (IL-6) type, LEPRb has three domains: an extracellular, a single membrane-spanning and an intracellular domain. Although LEPRb lacks intrinsic enzymatic activity, it interacts to the Janus kinase 2 cytoplasmic tyrosine kinase (JAK2) [147, 148]. JAK2 activation and autophosphorylation on many tyrosines are induced by leptin [149]. Additionally, LEPRb is phosphorylated by JAK2 at tyrosine residues 985, 1077, and 1138 [150]. Tyrosines phopho-Tyr985, phopho-Tyr1077, and phopho-Tyr1138 act as binding sites for downstream signaling molecules with the Src homology 2 (SH2) domain, attracting these molecules to the LEPRb-JAK2 complex where JAK2 phosphorylates these effector proteins [151].

### Leptin signaling (positive view)

#### LEPRb Tyr^1138^-streamed JAK2/STAT3 signaling

JAK2 phosphorylates LEPRb on Tyr 1138 in response to leptin, and phospho-Tyr1138 binds to the SH2 domain of signal transducer and activator of transcript 3. (STAT3). JAK2 connected to LEPRb subsequently phosphorylates STAT3, leading to dimerization and nuclear translocation [151, 152]. Its target genes, such as suppressor of cytokine signaling 3 (SOCS3) and neuropeptides, regulated by STAT3 dimers which function as a transcription factor in nuclei [153, 154]. Genetic studies have shown that leptin's ability to reduce obesity depends on the JAK2/STAT3 pathway [155].

#### LEPRb Tyr^1077^-streamed JAK2/STAT5 signaling

Leptin induces Tyr1077 phosphorylation on LEPRb, and phospho-Tyr1077 binds to STAT5's SH2 domain, enabling JAK2 to phosphorylate and activate STAT5. In addition, phospho-Tyr1138 helps STAT5 become activated [156, 157]. Obesity and hyperphagia are the outcomes of STAT5 removal from the CNS [158].

#### LEPRb Tyr^985^-streamed SHP2/ERK signaling

Tyr985 is phosphorylated to give the protein tyrosine phosphatase 2's SH2 domain a place to attach (SHP2). Leptin stimulates the extracellular signal-regulated kinase (ERK) pathway, which is mediated by SHP2 [159, 160].

#### IRS/PI3K signaling

Leptin activity also depends on the insulin receptor substrate (IRS)/phosphoinositide 3-kinase (PI3K) pathway. In hypothalamus, leptin activates the IRS/PI3K pathway. Obesity is caused by any obstruction in the IRS/PI3K pathway [161, 162]. Below is a description of two PI3K cascades downstream events:

A. The forkhead box O1 (FoxO1) signaling: FoxO1, an essential transcription factor for nutritional homeostasis, is a significant downstream effector of the PI3K/Akt pathway [163]. FoxO1 is retained in the cytoplasm and rendered inactive as a result of Akt's numerous phosphorylations of FoxO1 [164].

B. The mammalian target of rapamycin complex 1 (mTORC1) signaling: Another downstream process of the IRS/PI3K pathway is the mTOR/S6K pathway. The mTOR complex 1 (mTORC1) is stimulated by leptin to become activated, which phosphorylates and activates S6K in the hypothalamus and reduces appetite and body weight [165, 166].

### Leptin signaling (negative view)

The main risk factor for the development of overweight and obesity is thought to be leptin resistance. Leptin resistance is anticipated to be the result of deficiencies in any one of the LEPRb signaling cascades. Numerous intracellular proteins, such as SOCS3, protein tyrosine phosphatase 1B (PTP1B), and T cell protein tyrosine phosphatase, negatively inhibit leptin signaling (TCPTP) [167]. A leptin-targeted gene called *SOCS3* offers a negative feedback regulatory mechanism to stop the LEPRb pathways from becoming overactive [168]. Through direct binding to JAK2, SOCS3 reduces the activity of the JAK2 kinase [169]. In addition to binding to phospho-Tyr985, as previously mentioned, SOCS3 also prevents LEPRb signaling [168]. A class 1 non-receptor protein tyrosine phosphatase called PTP1B dephosphorylates and prevents JAK2 from functioning [167]. When the PTP1B gene is deleted from neurons, less calories are consumed and more are expended [170]. STAT1 and STAT3 are dephosphorylated by TCPTP, a different non-receptor PTP [171]. Leptin sensitivity is increased by inhibiting neuronal PCPTP, and brain elimination of PTP1B and TCPTP has an additive impact [172].

### Leptin and immune system

Modifications in an organism's metabolic state, such as the emergence of obesity or underweight as a result of excess or inadequate nutrition, can affect the metabolism of a single cell [173].

T cell metabolism relies on the cell activation status. Following activation as a result of increasing energy consumption, effector T cells, switch from mitochondrial pathways to glycolysis. This activation in CD4 + T cells is mediated via Akt and STAT5 intracellularly, signaling the increased glycolysis. Glycolysis mediates the rapid generation of Th1 and Th17 inflammation and promotes the production of IL-2 and IFN-γ. The initiation status of the cell affects T cell metabolism just like it does for other immune cells. In contrast, effector T cells convert from mitochondrial to glycolytic pathways in response to the rapidly rising energy demand after activation. Expression of genes associated in glycolysis and glutaminolysis is increased following T cell receptor (TCR) stimulation [174, 175]. Akt and STAT5 signaling, by which glycolysis was increased, mediates the shift from the dormant state in CD4 + T cells intracellularly [176]. Glycolysis essentially stimulates the rapid synthesis of Th1 and Th17 inflammation and encourages IL-2 and IFN-γ production. whereas Th2 cells initially are dependent on glycolysis, increased lipid metabolism pathways, such as fatty acid oxidation, synthesis, and absorption, are crucial for late activation and tissue adaption [177].

The Th2 cytokine-related genes expression of IL-5, GPR55, and ELAVL1 is upregulated in peripheral blood mononuclear cells (PBMCs) of asthmatics. Leptin present in breast milk activates leptin receptors in the baby's body, it activates the PI3K-AKT pathway and plays a role in suppressing Th2 production through mTORC1 [178].

### Intrinsic regulation

#### mTOR

The serine/threonine kinase mechanistic target of rapamycin (mTORC) 1 interacts with mTOR regulatory-associated protein (RAPTOR). mTORC1 largely incorporates signals that point to optimum conditions for cell development. mTORC1 senses the availability of nutrients [179].. mTOR is a PI3K downstream target that is essential for controlling cellular metabolism globally. A kinase known as PI3K is triggered by substances that affect both cellular metabolism and cell proliferation, such as leptin and the epidermal growth factor [180]. This permits T cells to increase catabolic pathways, including glycolysis and lipolysis, and fulfills the high energy requirements of effector responses after activation [174]. mTOR controls T cell development, proliferation, metabolism, and differentiation in response to changes in the availability of growth factors and nutrients. While mTOR inhibition leads to primary differentiation into Tregs, mTOR activation is a crucial prerequisite for the development of naive T cells into Th1, Th2, or Th17 cells. T cells have less ability to proliferate when mTOR signaling is absent [181]. The inhibition of autophagy by mTORC1 activation results in increased glycolysis and lipid synthesis pathways [182]. Th2 differentiation can emerge in the absence of mTORC1 but not mTORC2, and is more responsive to graded reductions in mTORC1 activity. Growth factors, oxygen availability, and amino acid availability are among the signals that mTORC1 responds to, whereas mTORC2 largely responds to other stimuli. However, it has been demonstrated that mTORC2 signaling reacts to nutritional variations, and only stimulates Th2 cell differentiation, not Th1 or Th17 [183, 184]. These findings support the idea that Th2 differentiation is less reliant on nutrient-sensing mTORC1 signaling and is instead more dynamically regulated by both nutrients and cytokines/growth factors. Activation of the PI3K pathway upstream of the mTOR-signaling enhances mTORC1 activity through AKT, whereas mTORC2 regulates AKT concentrations, and thus modifies mTORC1 function. Obesity increases glucose availability because of the increased dietary absorption, which in turn causes an elevation of glycolysis and the stimulation of Th1 differentiation of T cells [185].

#### AMPK

The AMP activated protein kinase, or AMPK, responds to the energy level of the cell by detecting the ATP: ADP ratio, controls glycolysis in accordance, and contributes to the regulation of both T cell activation and the cell's energy homeostasis [186]. When the cell is in a low energy state, indicated by low ATP levels, AMPK is activated, inhibiting mTORC1 and downregulating glycolytic pathways, Th1 and Th17 inflammation, and other processes [187]. Contrarily, AMPK stimulates mTORC2, which in turn encourages the breakdown of fatty acids and the Th2 and Treg immune responses [188]. Th1 cells primarily upregulate glycolysis to produce energy, with the help of mTORC1 signaling, which can phosphorylate the Tbet transcription factor directly in the Th1 cell [189]. Together, mTORC1 and mTORC2 boost type 2 inflammatory responses, and AMPK reduces type 1 inflammatory responses by blocking mTORC1 [185].

### Extrinsic regulation

#### Leptin

Adipocytes release the pro-inflammatory adipokine leptin which controls glucose metabolism and food intake to control energy conversion and consumption [190]. When leptin binds to the leptin receptor in the brain, it can cross the blood–brain barrier and start a number of signaling cascades that has an impact on how much food is consumed and how the body balances its energy. Reduced leptin signaling in the brain is caused by impaired leptin blood–brain barrier crossing, dysfunctions in the downstream pathways, or decreased sensitivity of the leptin receptor. These are potential causes of leptin resistance, which may then result in an imbalance in the energy homeostasis [191]. Leptin has also been demonstrated to support T cell survival and expansion [192]. Leptin resistance may develop when the serum leptin concentration rises during obesity. The obesity phenotype may become even more severe as a result of the increase in food intake, decreased nutritional absorption, and suppression of lipid and glucose metabolism [193]. Leptin increases IFN-γ release and inhibits Th2 in people, especially those who are obese [194]. The leptin receptor is not expressed by naive T cells, but it is upregulated after activation. Leptin increases glucose uptake and metabolism in activated CD4 + T cells, which increases the Th1 [195] and Th17 [196] cellular response while suppressing the Treg cell population [197]. In fact, a different study found that leptin inhibited IL-4 synthesis while increasing Th1 cytokine production [198].

**Fig. 1:**
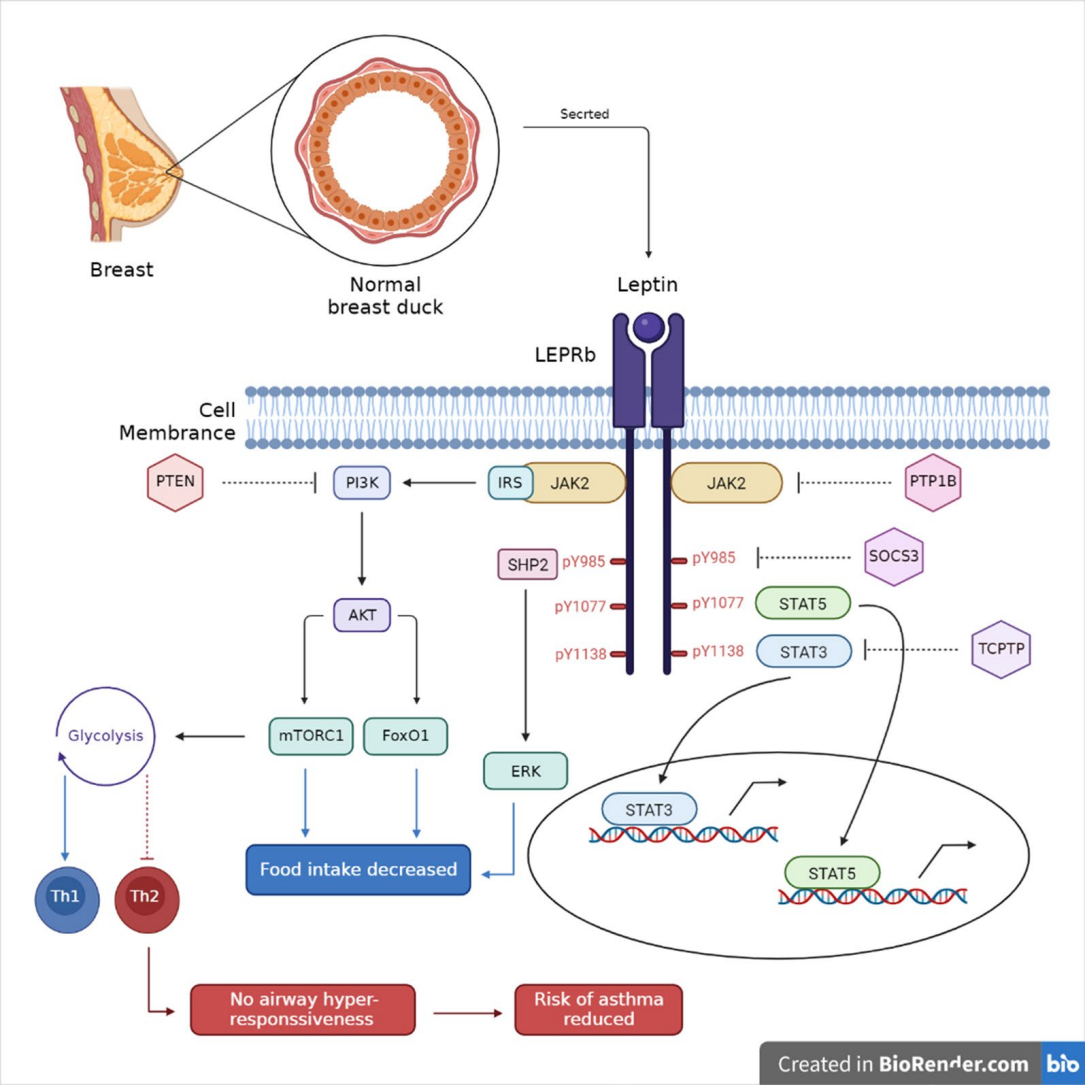
Molecular pathways of leptin in obesity-related asthma development

Figure 1 summarizes the underlying mechanisms involved.

## Conclusion

Breast milk has significant effects on infants in their early years of life which can protect their bodies against infections, developing asthma and being obese in the future. Obesity and asthma are shown to be linked conditions in childhood.

According to studies, obesity causes or exacerbates asthma symptoms, through a variety of mechanisms including increased inflammatory cytokines, reduced lung volume, and disorders like GERD and OSA. On the other hand, children with asthma are at risk for obesity due to inadequate mobility and the use of corticosteroids. As a result, it is critical to plan ahead and have a strategy in place to deal with childhood obesity and asthma. Although existing literature provides explanations for the protective role of breastfeeding against the two conditions, other factors such as duration of breastfeeding, parental, and prenatal factors may confound this relationship which require further research. Future studies are recommended to evaluate effects of breastfeeding in asthma and other allergies prevention in obese and also normal weight children.

## Data Availability

Data sharing is not applicable to this article as no datasets were generated or analysed during the current study.
